# Adoption of telehealth technologies: an approach to improving healthcare system

**DOI:** 10.1186/s41231-022-00125-5

**Published:** 2022-08-09

**Authors:** Arpana Sharma, Madhu Pruthi, Geetanjali Sageena

**Affiliations:** 1 Department of Mathematics, Keshav Mahavidyalaya, University of Delhi H-4-5 Zone, Pitampura, Delhi, 110034 India; 2 Principal, Keshav Mahavidyalaya, University of Delhi, H-4-5 Zone, Pitampura, Delhi, 110034 India; 3 Department of Environmental Studies, Keshav Mahavidyalaya, University of Delhi, H-4-5 Zone, Pitampura, Delhi, 110034 India

**Keywords:** Telehealth, Pandemic, Sustainable development, Policymaking

## Abstract

**Background:**

Globally, the healthcare industry is well known to be one of the strongest drivers of economic growth and development. The sector has gained substantial attention to deal with the fallout of COVID-19, leading to improvement in the quality observed in developed and developing nations. With the advent of the twenty-first century, globalization an ever-growing populace, and environmental changes prompted the more noteworthy spread of irresistible diseases, highlighting the association between wellbeing and future health security. The massive spread of COVID-19 paralyzed the global economy and took a toll on health governance and wellbeing. The present review aims to map the harrowing impacts of COVID-19 on the QoL (quality of life) observed. Particularly the post-pandemic era is likely to boot-strap the healthcare sector. Hence in post COVID era, there is a dire need to strengthen the healthcare system and understand the evolving challenges to answer calls in recovery in the wake of COVID-19.

**Conclusion:**

There is a flurry of research highlighting the implications faced due to the rise of the pandemic, resulting in the wrecking growth and development. However, the massive potential of telehealth is still largely underexplored with scarce research on countless evolving technologies. The current crisis highlighted the need to develop emerging frameworks and facilitate multilateral cooperation. The present research can serve as the baseline for better future strategies to improve global health initiatives. Further, this can help to focus on wider health determinants, redesign strategies and policies for the healthcare industry and to mitigate/deal better with future pandemics.

## Introduction

The viral disease COVID-19 caused by SARS-CoV2 (Severe Acute Respiratory Syndrome Coronavirus 2) first arose in the Hubei region of China in December 2019, spreading quickly worldwide has emerged as a global pandemic [1, 2]. The WHO announced the COVID-19 episode as a health emergency of worldwide concern (http://www.who.int/csr/don/12-january-2020-novel-coronavirus-china/en/).

It has now been more than one and a half years since the first outbreak. Since December 2019, there have been 535,143,050 affirmed cases of COVID-19 (as per the applied case definitions and testing methodologies across the impacted nations) and have accounted for 6,328,694 fatalities (https://www.ecdc.europa.eu/en/covid-19-pandemic). Representing COVID-19 patients, India is too adversely affected and remains in the second situation right next to the United States of America (https://covid19.who.int/region/searo/country/in).

The dynamic population statistics mirror the potential drivers triggering the viral infection and sequelae of healthcare implications faced exerting a substantial healthcare burden. Notwithstanding its prompt negative well-being impacts, the pandemic will certainly prompt various long-haul medical conditions [3–5]. The human cost of COVID-19 is critical, yet its actual scale is uncertain. COVID-19 has likewise negatively affected the worldwide economy. The World Bank anticipated since World War II, the most noticeably global recession, with the expected derailment of the worldwide economy [6–8].

Despite the immense efforts, the lack of antiviral medications posed an unprecedented challenge, representing a dire need for current worldwide endeavors to contain the COVID-19 outbreak. The continuous viral genomic transformations remain an elusive concern for future resurgence [9–11]. The double trouble i.e. gripping of pandemic and ongoing resurgence has likewise put the global healthcare system at the tipping point. The nipping spread of the disease on a global scale demands the setting up of increasingly more COVID-19 healthcare facilities. Paradoxically, pandemic reaction plans across nations are often unable to address wellbeing prerequisites, necessities, and contemplations. However, Telehealth technologies appear to be a promising technique that might be utilized to deal with the ongoing health crisis. The COVID-19 act as a wake-up call to a multipronged approach that shall aid in the reorganization and strengthening of the present healthcare system. The adaption of the technologies shall pave the way, in the long run, mitigating the dramatic impacts of the crisis and accomplishing the dream of a COVID- free future.

## Telehealth technologies and health access durinG COVID-19

The ICT (information and communication technology) has expeditiously transformed the healthcare environment, telehealth has drawn developing interest from both consumers and healthcare experts. Telehealth, a more extensive idea than telenursing or telemedicine, is defined as “the use of telecommunication technologies and computers to exchange health care information and to provide services to clients at another location” [12]. Telehealth services are being provided to registered medical practitioners, including psychotherapists, social workers, nutritionists, and nurses, in various care settings for disease management [13, 14]. A consensus has been reached for the successful utilization of telehealth in alleviating persistent/chronic health issues [15–17]. This is relied upon promptly to be an efficient, cost-effective remote management with increased patient outcomes. A plethora of studies found favorable clinical outcomes from the telehealth approaches [18–20]. Yet, the telehealth interventions, implementation, and outcome measures are not conclusive [21].

The cascading COVID-19 pandemic has set off a quick shift in reigniting telehealth from conventional healthcare [22–24]. Telehealth advances have been expanded during general wellbeing crises, particularly during the spectrum of natural or anthropogenic disasters [25]. Telehealth is the modus operandi of the twenty-first century which permits effective screening of the patients; with patient-centric considerations provided and helpful for the management remotely. This methodology ensures to reduce the physical exposures and thus controlling the viral spread among the populations including patients, clinicians, and other stakeholders. The approach hence aids to a stage where doctors and patients both can cooperate with no constraints of time and day utilizing cell phones and/or webcam-empowered PCs.

Quite a few studies have added to the utilization of telehealth and given directions on the best way to combat the deleterious impacts in response to COVID-19 [26–28]. The quick take-up of telehealth has for the most part been because of the need to follow the physical distancing prerequisites and the need to diminish the danger of transmission. Even though telehealth has been accessible for a long time, the COVID-19 experience has brought about uplifted familiarity with telehealth among wellbeing specialists, patients, and society in general. Nonetheless, the innovation program of telehealth cannot be made short-term, in addition, the exhaustive telehealth development can be beneficial yet pose certain challenges to sustainable transformation [29, 30]. Furthermore, the telemedical advancements executed in developed nations like the United Kingdom and the United States of America must be used to improve COVID-19 outcomes [31, 32]. The analysis trends observed in the USA recommend that the fast expansion in telehealth that happened during COVID-19 is currently consistently declining (https://www.statnews.com/2020/06/25/telemedicine-time-to-shine-doctors-abandoning-it/). Partially this is reasonable because of sure in-person benefits which were initially required to be postponed during the reoccurrence of COVID-19 since the beginning stage of the coronavirus pandemic. Another explanation is that clinicians might be lessening their utilization of telehealth because they needed earlier preparation and were not prepared to take on telehealth in their clinical practice [33, 34].

Coronavirus helps us to remember the many connections among wellbeing and manageability challenges and the need to pivot on associated tradeoffs during/ after recovery. Along these lines, the usage of telehealth applications during the current pandemic is essential to assess the worth of telehealth the current pandemic. With expanded numerous telehealth take-ups during the pandemic, it is opportune for future healthcare deliveries and essential to consider the precise role of telehealth in the post-pandemic era. The myriad of telehealth applications and their implementation could suggest a roadmap for its far-reaching potential in planning, framework designing, and policymaking amidst debacles/crises. Thusly, it is vital to redesign the healthcare system considering the crucial role of telehealth in the medical sector. The long-term advantages shall trump the pathways to arrive at concurred objectives to promote health and wellbeing.

## Roadmap to success of the telehealth technologies

There has been a fast take-up of telehealth, remembering for settings where this was thought to be infeasible. [35] investigated the job of telehealth as a fundamental instrument of medical care administration conveyance, particularly during the pandemic. Thusly, the creators assess the security and viability of telehealth visits in furnishing patients having to post neurosurgical consideration. The said discovery prompted the quick development of telehealth in dealing with the necessities of the neurosurgical patient populace. In light of their examination, the utilization of telehealth radically expanded across the domains of neurosurgery, with a huge increment in first-time online experiences, to address the patient’s issues. The telehealth market is exploding and is expected to grow more. The utilization cases are moving from distant patient consideration through information sharing, to distant care and wellbeing through live association and teleconsultation. For instance, In Germany, the telemedicine law (2018), which was already leisurely taken up practically speaking, has seen a quick reception during the peak of the coronavirus crisis (https://www.aerzteblatt.de/nachrichten/110997/Telemedizin-Kraeftiger-Schub-fuerVideosprechstunden). In Canada, new charge codes for virtual consideration were optimized promptly while earlier these sorts of subsidizing choices were restricted (https://www.thechronicleherald.ca/lifestyles/regional-lifestyles/virtual-care-use-growing-in-nova-scotia-amidst-covid-19-precautions-432977/). Virtual consideration likewise supplemented the arrival of retired folks as telehealth was viewed as a promising route for more established clinicians to all the more securely patient-care (https://www.statnews.com/2020/03/25/protect-older-and-vulnerable-health-care-workers-from-0covid-19/). Despite numerous expectations about the development in telehealth, it is as yet not considered to be a standard activity in the healthcare sector. It has filled in significance and use, yet the prevailing working or plan of action is the in-person/face-to-face patient consideration.

The more extensive utilization of telehealth has uncovered significant gaps in the preparation of healthcare services to convey telehealth on a routine clinical basis. These incorporate the knowledge, capacity-building gaps inside the current well-being workforce, lack of funding plans, restricted information, and frameworks in sharing challenges (https://croakey.org/unpicking-some-key-challenges-for-the-telehealth-revolution-ahead/). Solutions are needed to lessen the rising issues in a way that the healthcare sector can suitably further plan its administrations.

**Fig. 1:**
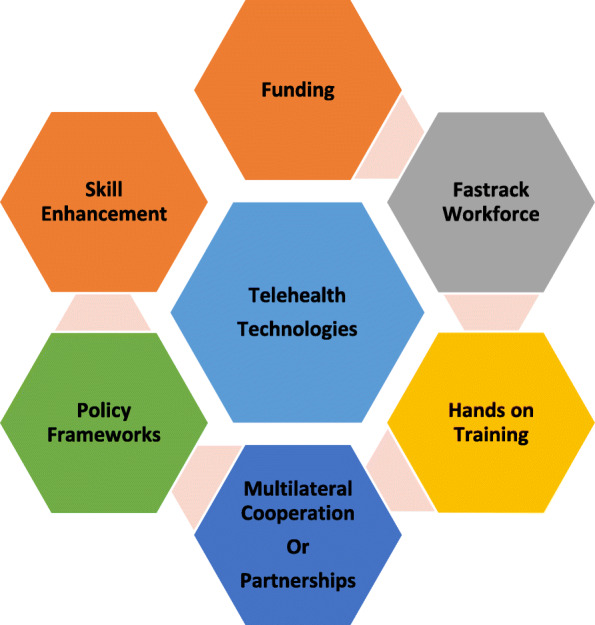
A Roadmap to the success of Telehealth Technologies

Figure 1 highlights the roadmap to the success of telehealth technologies. The developing pattern in medical services overall is preventive, prescient, customized, and participatory and these patterns are precisely increasing the digitization of medical services during the COVID pandemic [31]. Telehealth programs could stamp out the actual obstructions to provide patients with helpful clinical consideration. Medical care frameworks with telehealth support the coherence of short-term patient consideration during this pandemic amidst “remain at-home” orders and physical separating measures, while diminishing nosocomial and local area spread. Telehealth likewise demonstrates valuable for ongoing consideration, specifically to assist balance the inventory of clinical administrations with flood sought after across physical or topographical limits and in providing confined patients association with loved ones. While talking about the roadmap to success of telehealth technologies. Considering telehealth requires extra abilities and proper support [36]. COVID-19 has featured that an enormous extent of the labor force has not been prepared on how to convey care utilizing telehealth [37]. The fast pace of telehealth requested imaginative models of preparation and support to guarantee the staff fostering the important abilities to convey telehealth administrations. Admittance to progressing specialized help and preparation is important to help staff with the utilization of telehealth innovations will be an optimal chance in preparing and guaranteeing the utilization of best healthcare practices. Improvement in abilities through reasonable capabilities and authorization will fortify the things to come. Telehealth capabilities ought to be needed to guarantee the current practitioners keep up with their abilities and the arising workforce to foster those abilities. Given the involvement of telehealth observed amidst the pandemic, numerous buyers might anticipate telehealth to be a choice specifically as an enhancement to in-person visits or for low-acuity issues. Reckoning consumer’s assumptions and figuring out what kind of care probably going to be beneficial is significant. To diminish COVID-19 transmission and empower distant care to be given, might be accomplished through cognizance of multilateral cooperation. Policymakers comprehend a pertinent need for a mixture of innovation in the medical care area making it more useful, broadening the net to build access, and increasing the last mile conveyance (https://www.business-standard.com/article/pti-stories/doctor-patient-ratio-in-india-less-than-who-prescribed-norm-of-1-1000-govt-119111901421$_$1.html). The COVID-19 flare-up and resulting blast of telehealth administrations presents an unrivaled chance to analyze related issues during a period of emergency in medical services, and later. The scope of issues and current encounters propose an entirely new framework technique" to implant telehealth into routine assistance and other data framework capacities. Doing such requires data innovation organizations, approaches, methods, mechanical framework, and viable changes to the board procedures for new work processes and care models This is critical to remember patients for the telehealth technologies plans to guarantee that they honor the clinical results and more individual care [38]. Calls for supported funding are mostly lined up with the continuation of impermanent COVID-19 changes (https://www1.racgp.org.au/newsgp/professional/expanded-access-to-telehealth-could-continue-after). Since telehealth is a significant well-being innovation arrangement that has acquired a further foothold during the COVID-19 pandemic [39, 40]. There is by and large expanded interest for telehealth administrations across the fifty nations generally impacted by coronavirus disease, featuring the requirement to increase telehealth capacities, during and past the pandemic [41]. These discoveries highlight a squeezing need for strategy creators and medical services suppliers to increase telehealth foundation and ability, during and past COVID-19.

## The possible nexus of sustainable development, health and technology

-The vision of the United Nations Sustainable Development Goals [42] has expansively bolstered communities on a global scale. Progress toward the objectives before the emergence of the ongoing health crisis was very sluggish, and the pandemic has ushered tremendous extra boundaries as economies derailed and disparities exacerbated [43, 44]. Notwithstanding, it would be a misstep to presume that the SDGs are in some ways or another less important at this point. However, one wouldn’t need to wait for a pandemic to reflect on the benefits of telehealth technologies. The SDG system remains to trump for recognizing the robustness and coordinated efforts to secure the current health of the current as well as the future while limiting pessimistic compromises during the COVID-19 emergency and resulting recuperation [45]. The Coronavirus pandemic is indeed the best worldwide emergency that consistently occurred. Not just health-related problems and economic, social, and policy-driven issues are completely affected by this pandemic. Further advancement in targets of SDG-3 (Good Health and Well-being) might have decreased pressure on delicate wellbeing frameworks troubled by significant degrees of transferable infection; diminished the danger of extreme COVID-19 results related to non-transmittable illnesses like diabetes, respiratory sickness, and chronic, kidney disease [46]; and reinforced wellbeing frameworks and the limit concerning early warnings and better governance of worldwide health risks. The current social and financial well-being imbalances have intensified the adverse consequences of the pandemic. Expanded travel and tourism industry frameworks are one of the primary human-intervened drivers of the rise and spread of zoonotic sickness [47]. Then again, progress toward different objectives might have exacerbated the pandemic, building the danger of the following zoonotic sickness flare-up [48]. The development of roads and expanded network to distant regions (connected with SDG 9) is probably going to build the chances of irresistible illnesses [49]. The SDG system features where our reaction to ensuring wellbeing during the coronavirus health crisis has hampered progress towards different objectives, for instance, the enormous expansion in single-use plastics, the greater part of which will end up on land-based and water-based ecosystems (connected with SDGs 12 and 14). One evaluation found that accomplishing eighteen percent of SDGs targets may decrease the danger of new infections pouring out from newer virus strains to people, especially targets connected with illegal trading of wildlife products and reducing trafficking [48]. As needs are, accomplishing the 2030 Sustainable Development Goals is a major cross -swords in wake of the COVID-19 pandemic. Accordingly, nations that acknowledged the 2030 SDGs should keep ensuring feasible medical services arrangements [50].

Undermining the state of affairs can move power structures and propel entertainers to stray forcefully from present plans and approaches [51]. Although the fact that there is a possibility of precarious bouncing back to the same old thing, COVID-19 ought to be taken as an advantage towards avenues of change. The economic fallouts of the pandemic should trigger approaches and moving standards that could convey de-carbonization and progress towards other SDGs. The most encouraging and fundamental will be the empowerment of public support. The pandemic has provided us with a few instances of successful initiative and correspondence methodologies to fabricate support for troublesome measures in light of a legitimate concern for the benefit of everyone. The worldwide reaction to Sars-CoV-2 has been depicted as the ’greatest science policy failure of our generation [52]. Nations like Germany and New Zealand have remarkably used elimination policies to contain coronavirus and protect populations in nations [53, 54]. These experiences could be significant for shaping standards and support building to handle such emergencies.

**Fig. 2:**
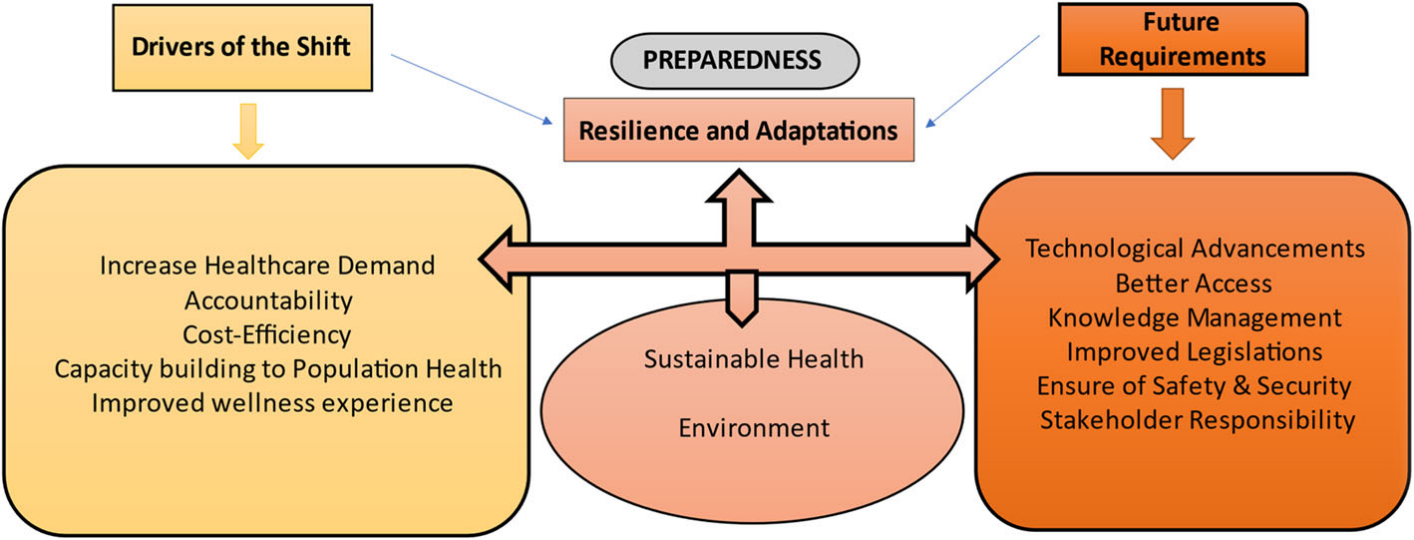
Coherent Strategy to implement Telehealth Technologies

Figure 2 describes the strategic planning for the proper implementation of innovative telehealth technologies. In response to the major drivers of the shift, research coordinated efforts and information sharing during the COVID-19 pandemic have led to the need to foster topical areas. Aiming at the recuperation and preparedness for future pandemics “sustainable health and environment” are expected to be constructed as a part of preparedness/future planning. The pith of nexus arranging is to rearrange human comprehension of the complicated interrelationships between these two constructs. The synergism of the two elements shall aid in alluding to the intricate connections between environment, wellbeing, and nutrition. The emanating impacts of health are intricately linked with the environment. Each of the conventional and current perils is related to an assortment of parts of social and economic turn of events. Besides, there is no single most effective way of getting sorted out and seeing the said relationship that uncovers all of the significant associations and conceivable passage focuses for general wellbeing targets. It very well may be contended that telemedicine was fast forward-thinking and that medical care is changing such that it can at last benefit from its capacities. As, medical services keep on moving from exchange-based to esteem-based, wellbeing frameworks have embraced the concurrent quest for further developing the patient-doctor consideration experience, even while combating coronavirus! [55, 56]. This shift to responsibility, cost-proficiency, populace wellbeing, and care experience is driving the fast reception of telehealth across the medical care industry [57–60]. The development of virtual medical services isn’t just expanding doctor access and driving down costs, but on the other hand is setting out open doors for innovation organizations to give quicker, more helpful patient encounters – bringing about further developed results and an upgraded experience [61–63]. The wider adoption of artificial intelligence- enabled telehealth can assist in the quality improvement of existing clinical practices [64, 65].

Legislative mandates have since quite a while ago affected the conveyance of telehealth drives, because the significant reception of telehealth regularly lies in legal language. While completely embracing telehealth under existing state and government arrangements ought to be needed, new approaches should address developing worries connected with repayment arrangements and permitting laws [66, 67]. Telehealth is essentially a methodology of conveying care. Thusly, the norm of care for each sort of administration actually applies, however, some contend there are no protective shields [68]. The security issues of telehealth applications and the utilization of media transmission straightforwardly influence the information security of patients [69–72]. Protection and security are the principle factors influencing the choices of consumers to embrace and use innovation [73]. Keeping up with high protection and security frameworks will build individuals’ trust and, henceforth, increment their acknowledgment to utilize telehealth administration. In any case, as the utilization of telehealth keeps on developing, the norm of care for telehealth experiences will probably advance concerning information assurance and keeping up with patient protection. In particular, main thrusts/drivers make the conditions in which environmental health can create or be turned away or that are created enormously by individuals in the quest for the essential necessities of life and an extent relying upon the sort of peril to which individuals have been uncovered. The strategic framework is expected to feature the significant connections between various viewpoints of holistic development and to help recognize successful arrangements and activities to control and forestall wellbeing impacts to be utilized in the current circumstance of human health and wellbeing.

## Conclusion

The key qualities of our review incorporate a remarkable approach to assessing the public interest for telehealth administrations, apprehending the contemporaneous responses in traction with the COVID-19 pandemic.

Telehealth can become an increasingly valuable resource in the healthcare industry providing promising results and decreasing expenses. It has progressed significantly and can possibly on a very basic level shift how one will access and provide medical services. As the adoption of telehealth keeps on developing, so will its associated challenges. The medical care industry needs to keep distinguishing the right patients, innovation, and other advancements cycles that will lay out the groundwork for telehealth. A study stated, that telehealth support has limited a portion of the necessary transformations and constraints, speeding up the fast development of telehealth [74]. The rapid increase in the applications of telehealth and endeavors during the ongoing situation of the health crisis, just as the vulnerability concerning pandemic, is relied upon to grow the capacity of telehealth. The utilization of telemedicine innovation can help specialists in patient care. It seems we are “going virtual” during an emergency, nevertheless, we must remember the most fundamental periods of change and how to convey/deploy telehealth at its best viably. Whether or not medical services undertakings are prepared, the real truth is that virtual considerations have proven. However, the absence of medico-legitimate considerations to help telehealth is a challenge in gaining the momentum of telehealth apart from the lack of understanding and experience with technical skills [75, 76]. Within the sight of a profound observation, the tailored health infrastructure requires the progressive adoption of digital technologies. Particularly the sacrosanct healthcare frameworks should attempt to strengthen ensuring a feasible telehealth foundation while considering the proficient utilization of emergency clinic space and staff. The coordination of telehealth necessities during the pandemic and the approval of telehealth advances are the subsequent stages to work on the feasibility and appropriate execution of the framework. An appropriate mix of telehealth as upcoming innovation to help patients, medical care suppliers, and policymakers can work on existing applications and advance the improvement of a clinical medical services practice to the best expectations. Further examination is expected to build the adequacy of telemedicine for better clinical practice later on. With the progress to a post-pandemic stage, the vital change of telehealth frameworks is to move from emergency mode to maintainable, secure frameworks that appropriately safeguard patient protection and information security sustaining and extending specialized support for post-crisis care.

## Data Availability

Not Applicable.
